# Midwifery, and Diseases of Women and Children

**Published:** 1843-01

**Authors:** 


### MIDWIFERY, AND DISEASES OF WOMEN AND CHILDREN.

### *Operation of Pelviotomy.* By Dr. IPPOLITO.

THIS operation was performed in the Ospedale degl'Incurabili. The patient was twenty-three years old, and rickety; she was nine months pregnant, and three days before her admission, the liquor amnii had escaped after slight pains which had continued ever since. The pelvis, measured with a Baudelocque's pelvimeter, had a sacro-pubic diameter of three inches, but the base of the sacrum was much inclined to the left, so that the distance from it to the ilio-pubic symphysis of the same side was not more than one inch, and the whole left half of the cavity of the pelvis was entirely useless for parturition. The head presented, but the os uteri, after four days' pains, was scarcely enough dilated to admit a finger. After a consultation of all the professors, it was determined to perform pelviotomy according to the method of Professor Galbiati, who was himself present. He would have waited till the os uteri was somewhat dilated in order that directly after the operation the fœtus might be extracted alive by the forceps, or by turning; but the others were for immediately operating.

Dr. Ippolito therefore proceeded at once to the operation. It was long but well borne, and not an ounce of blood was lost. [The details are not given; but we gather that the bodies of the ossa pubis, and the ascending rami of the ischia were cut through with strong-toothed forceps.] After the operation the patient was put in a warm bath, and some anodyne medicine was given, which refreshed her. In the following night the labour-pains increased, and in the morning they were powerful; but the orifice of the uterus was not much dilated. Ten grains of ergot of rye were given; then a tepid bath, and then another dose of the ergot. The pains were thus increased, and became very frequent, and a little before noon a well-nourished and large child was born; but though it had given distinct signs of life while in the perineal space, it was now asphyxiated and could not be recovered.

After the birth the mother's pulse, which had been small and contracted, rose, and some hours after she had fever with a tendency to vomit; but after a calming draught she passed the whole night quietly. On the following day she had slight fever, the uterus was contracted and without pain, the lochia regular, the abdomen soft and without pain; only she complained of a pain in the right iliac region. A bath gave some ease, and castor oil was administered with effect. All the rest of the day she had flatulence and eructation with tendency to sleep and slight fever. On the morning of the second day after the operation she was better, and had had several evacuations from the bowels: the pulse was eighty-four; the tendency to vomiting and the sleepiness had ceased; the abdomen remained soft. A bath and magnesia-water were prescribed. On the third day the abdomen was tense and there was meteorism; the pulse was ninety-two. Twenty leeches were applied to the hypogastrium, and other means were employed with some temporary relief, but next day she was much weaker, and the peritonitis had increased. She died on the sixth day after the operation.

At the autopsy, the wounds were found gangrenous. There were spots of ecchymoisis on the outer part of the uterus, with a purulent albuminous fluid, opposite the wounds through the pubis. The uterus was of the size usual in that period after parturition: there was some redness with a little purulent effusion around the right round ligament. All the peritoneum was reddened; the intestines and other organs were natural; the mucous membrane of the uterus was as if gangrenous, its parenchyma healthy but congested. There was slight purulent effusion in the iliac fossa.

The reporter, a pupil of Prof. Galbiati, remarks, that the operation might have been shortened by using a chain-saw instead of the forceps for cutting through the bones: and that it was performed too soon. By waiting till the os uteri was dilated, the patient, weakened by the operation, would have been spared about twenty hours' labour-pains.

*Il Filiatre Sebezio. Marzo,* 1842.

---

*Account of a Case in which an extraordinary number of Worms was voided by a Child.* By Dr. Gilli, of Turin.

A boy, the child of healthy parents, always had good health till he was weaned when fourteen months old. Soon after this period he had an attack of gastro-enteritis, which yielded, however, to antiphlogistic treatment, but was followed by the formation of a large abscess in the left axillary region. His illness appeared to have been in great measure induced by unwholesome food which his nurse had given him, and after his recovery his parents were surprised to see that though in other respects well he yet devoured with avidity, earth, chalk, and other indigestible substances. He was now eighteen months old, when symptoms of gastro-enteritis reappeared, for which local depletion and other antiphlogistic measures were had recourse to. Under this treatment the abdomen had become soft and the constipated bowels had begun to act, when suddenly the belly again grew tense and painful, and colic-pains came on with such violence, that the patient threw himself out of his cot and rolled in agony on the floor. He demanded food incessantly, and devoured whatever was given him. The eyes during this time were fixed, the pupils dilated, the tongue was red and loaded, and the breath fetid. With this was associated considerable disturbance of the nervous system with frequent convulsive movements of the limbs and twitchings of the muscles of the face. Fomentations were applied to the abdomen and vermifuge medicines were given. On the first day of their employment the patient voided twelve lumbrici. The symptoms continued unabated on the following day, the same remedies were persevered in with the addition of an enema, which produced the discharge of a great number of worms and manifest relief of the symptoms. Many of the symptoms, however, continued, and vermifuge remedies were accordingly persevered with, and with such effect that in the space of eight days 510 worms were discharged, some alive, others dead, most of them about six inches in length. Some of these worms were vomitted, but by far the greater number were discharged by stool, and the little patient was so exhausted by his efforts to get rid of them, that they were extracted as often as they made their appearance at the anus. At the end of eight days no more worms were passed, and the child rapidly recovered his health.

*Giornale delle Scienze Mediche di Torino. Marzo,* 1842.

---

*On Abdominal Apoplexy in New-born Children.* By Dr. Fr. von Kiwisch, of Prague.

This name is applied by the writer to a number of affections which are unnoticed by many writers, and by others are classed, though improperly, under the head of hæmatemesis, melæna, &c. The writer has observed apoplexy of the intestinal canal four times, and of the liver twice, as sole cause of death, and has observed it several times in the liver and once in the spleen in connexion with other diseases.

In the four cases of apoplexy of the intestinal canal, the children had attained the full term, and were born after easy labours. In two the umbilical cord was tied too soon, and in one it was found necessary to untie the cord, and to allow the escape of blood from it before respiration could be properly established. Within a few hours after birth, from twelve to thirty, the first symptoms of the disease appeared, the children discharging blood from the anus, and in two instances they likewise vomited it. The abdomen became distended and dull on percussion, the children, who at first were restless, grew by degrees quiet and pale, and they all died within forty-eight hours after birth with the signs of exhaustion from hemorrhage. After death all the organs were found remarkably bloodless, with the exception of the intestines and stomach, which contained a considerable quantity of blood. The effusion seemed to have taken place originally about the middle of the ileum, for here the blood was most completely infiltrated into the intestinal walls. The cause of this as well as of the other forms of abdominal apoplexy seems to be the tying the umbilical cord before the respiration is completely established. Whenever in a new-born child lividity of the body appears, the funis must at once be untied and blood allowed to escape.

The cases of apoplexy of the liver were marked by the children becoming restless and refusing the breast. On the following day the abdomen began to swell, and on the third day the children died. In one instance a considerable quantity of grumous blood was found in the abdominal cavity, the right lobe of the liver was much distended, and the peritoneum investing its lower surface formed a fluctuating pouch, in the middle of which was a laceration four lines in length, that gave exit to a considerable quantity of fluid blood. This sac was full of blood and softened liver, but elsewhere the substance of the organ was of natural consistence. In slighter cases there were merely slight extravasations of blood beneath the peritoneum, but not the substance of the liver.

The author only once met with splenic apoplexy, in connexion with a pneumonia, which proved fatal. In this instance the extravasation was seated in the lower part of the spleen where the parenchyma of the organ was quite broken down; its tissue elsewhere was healthy.

*Oesterreich. Medicinische Wochenschrift*, 1842. Nos. iv. & v.

---

*History of the Epidemic of Croup which prevailed in* 1840 *and at the beginning of* 1841, *in the Hôpital des Enfans, at Paris.* By M. E. BOUDET, formerly *interne* of the Hospital.

THE essay (which has been crowned by the faculty of medicine,) begins with a brief historical sketch of the various epidemics of croup of which authors have made mention. They may for the most part be referred to the class either of simple croup limited to the air-passages,—of croup attended with angina and membranous exudations,—or of croup accompanying exanthematous fevers, while a fourth division may include those epidemics of which the accounts in these respects are defective. It appears that croup, when epidemic, has usually coexisted with angina and membranous exudations; that once it was associated with gangrene of the pharynx; that it has but seldom accompanied the exanthemata; and that in some instances its primary seat has been in the air-passages.

A review of the cases of croup at the children's hospital from 1820 to 1839, follows next. It appears that the cases have been most numerous in the autumn months, least so in the spring, while their frequency has been just the same in summer as in winter. The age from two to five years is the most liable to the disease, and it occurs more frequently in boys than in girls. Nineteen of twenty-two subjects examined after death presented a false membrane in their air-passages, which extended into the bronchi, in seven cases, and to the pharynx only in two.

Such are the chief results afforded by the cases of sporadic croup observed during nineteen years. The number of cases which occurred during 1840 and the early part of 1841 was thirty-six, and the description of this epidemic forms the especial object of the paper.

The unusual prevalence of croup in 1840 was not confined to the Hôpital des Enfans Malades, but was likewise observed in the Hospice des Enfans Trouvés, as well as in private houses in Paris and at Montmartre. The first case of croup in the hospital in 1840, appeared in the month of March, the weather being cold, and the wind northerly. In the following three months four cases of croup occurred; four likewise in the succeeding three months; but in the last quarter of 1840, sixteen cases were admitted. The epidemic did not altogether cease at the close of 1840, but cases continued to occur during the following year. It was remarkable, however, that in no instance did the disease appear in an uncomplicated form, as it had done in the previous year, but always either accompanied or followed diphtheritis. Angina, with formation of false membranes, increased in frequency as the spring advanced, and became associated, in May, with a tendency to gangrene not only of the tonsils, but also of any part from which the skin had been removed by a blister, or sinapism. Croup now became more seldom, but again reappeared in June and July, and did not cease finally until towards the end of 1841.

The instances in which croup appeared wholly devoid of all complication were only four in number; usually it came on in the course of an exanthematous fever, or followed soon afterwards, or it succeeded to a pseudo-membranous angina, or was complicated with gangrene of the skin or the pharynx. The symptoms did not present any important variation from those usually observed, but the post-mortem appearances are described with a minuteness which renders the details very valuable. False membranes existed in some part of the air-passages, in twenty out of twenty-three fatal cases. They were found in the trachea quite as often as in the larynx, but were always thicker and more developed in the latter. In rather less than half of the cases they extended into the bronchi, and in one instance they occupied the bronchi alone, and did not exist in the larynx or trachea. The subjacent mucous membrane usually presented a bright red colour, but without any considerable softening or thickening. In two instances the mucous membrane in contact with the false membrane was ulcerated, and in one of these cases there existed likewise several small collections of pus in the submucous cellular tissue. Whenever croup had not proved very speedily fatal, emphysema, both vesicular and interlobular, was found after death, and usually in a degree exactly proportioned to the mechanical obstacles to respiration. Both lungs were almost always found to be inflamed, but the pneumonia was not usually extensive, seldom occupying more than the base and posterior half of one lobe. In no instance did M. Boudet find the lung in a state of purulent infiltration, and in many cases the pneumonia had not passed beyond the first stage.

The mortality of croup is very variously estimated by different writers, and the discrepancies between their statements are so great that one can hardly avoid the supposition that some essential difference must have existed between the diseases they observed. The late epidemic at Paris has been peculiarly fatal. Of twenty-five children received into the children's hospital labouring under croup, in the year 1840, twenty-three died, and of twelve received during the first six months of 1841 all died, or, in other words, the proportion of deaths has been about three times as great in 1840 and 1841, as in former years. In the city of Paris too there was a striking increase in the number of deaths from croup. The number who died from croup in 1838 was 187,

1839 .... 286,

1840.... 326.

The epidemic croup of 1840 coincided with an unusual prevalence of exanthematous fevers, raging as they became more frequent and subsiding with their decline; till, on the supervention of a pseudo-membranous angina in Jan. 1841, croup was again reexcited. It did not appear as it is usually stated to do at a time when bronchitis, pneumonia, and catarrhal affections, are particularly prevalent, but the very opposite of this occurred. From an examination of all the causes of the epidemic, M. Boudet assigns the first place to the exanthematous fevers, while the unfavorable hygienic conditions of children in the hospital probably increased their liability to the disease. The influence of atmospheric changes he regards as very doubtful, and the influence of contagion as still more problematical, except in 1841, when angina became very frequent, and was readily communicated from one patient to another.

In ten cases tracheotomy was performed, but life was not preserved in a single instance. The conclusions to which M. Boudet has arrived with reference to the operation will be best given in his own words.

"In conclusion," says he, "seeing that no advantage resulted from tracheotomy when false membranes existed in the bronchi, and that it appears to have given rise to ulcerations in one case, and to chronic inflammation of the trachea in another, seeing too that in every instance without exception in which it was performed, double pneumonia was found on examining the body after death, while in some cases of very severe croup in which the patients died after the disease had existed for some time, but in which tracheotomy was not performed, no inflammation of the lungs was discovered on a post-mortem examination, while on the other hand, in some instances where the disease was neither very severe nor very far advanced, life seems to have been prolonged, it may be fairly concluded that the advantage of tracheotomy were very small; that it apparently contributed to the production of pneumonia, and of ulceration or thickening of the trachea, and that when the bronchi contained false membranes, it was of no use whatever."

*Archives Générales de Médecine. Fevrier et Avril,* 1842.

